# The Comparison of the Multi-Layer Artificial Neural Network Training Methods in Terms of the Predictive Quality of the Coefficient of Friction of 1.0338 (DC04) Steel Sheet

**DOI:** 10.3390/ma17040908

**Published:** 2024-02-16

**Authors:** Tomasz Trzepieciński

**Affiliations:** Department of Manufacturing Processes and Production Engineering, Rzeszow University of Technology, al. Powstancow Warszawy 8, 35-959 Rzeszow, Poland; tomtrz@prz.edu.pl

**Keywords:** formability, metal forming, steel sheet, surface roughness, surface topography

## Abstract

Friction is one of the main phenomena accompanying sheet metal forming methods, affecting the surface quality of products and the formability of the sheet metal. The most basic and cheapest way to reduce friction is to use lubricants, which should ensure the highest lubrication efficiency and at the same time be environmentally friendly. Due to the trend towards sustainable production, vegetable oils have been used in research as an alternative to petroleum-based lubricants. The analysis of friction in sheet metal forming requires an appropriate tribotester simulating the friction conditions in a specific area of the sheet metal being formed. Research has used a special strip drawing tribometer, enabling the determination the value of the coefficient of friction in the blankholder zone in the deep drawing process. Quantitative analysis of the friction phenomenon is necessary at the stage of designing the technological process and selecting technological parameters, including blankholder pressure. This article presents the results of friction testing of 1.0338 (DC04) steel sheets using a strip drawing test. The experimental tests involved pulling a strip of sheet metal between two countersamples with a rounded surface. The tests were carried out on countersamples with different levels of roughness for the range of contact pressures occurring in the blankholder zone in the deep drawing process (1.7–5 MPa). The values of the coefficient of friction determined under dry friction conditions were compared with the results for edible (corn, sunflower and rapeseed) and non-edible (Moringa, Karanja) vegetable lubricants. The tested oils are the most commonly used vegetable-based biolubricants in metal forming operations. Multi-layer artificial neural networks were used to determine the relationship between the value of the contact pressure, the roughness of the countersamples, the oil viscosity and density, and the value of the coefficient of friction. Rapeseed oil provided the best lubrication efficiency during friction testing for all of the tested samples, with an average surface roughness of Sa 0.44–1.34 μm. At the same time, as the roughness of the countersamples increased, a decrease in lubrication efficiency was observed. The lowest root mean squared error value was observed for the MLP-4-8-1 network trained with the quasi-Newton algorithm. Most of the analysed networks with different architectures trained using the various algorithms showed that the kinematic viscosity of the oil was the most important aspect in assessing the friction of the sheets tested. The influence of kinematic viscosity on the value of the coefficient of friction is strongly dependent on the surface roughness of the countersamples.

## 1. Introduction

The metal forming processes of deep drawing high-quality steel sheets are among the leading methods of producing finished components, especially in car body production [1,2]. The mass production of car body parts takes place almost exclusively using sheet metal forming processes [3]. This method of plastic working is fast and does not cause production waste, which is inherent in machining processes [4]. The properties of the finished product, such as the surface quality, are mainly influenced by the friction and lubrication conditions [5]. During the deformation process, friction causes a change in the surface topography and material flow character of the deformed workpiece and a deterioration in the surface quality of the product [6,7]. About 15–25% of total energy consumption worldwide is used to reduce friction in mechanical systems [8]. Friction is particularly important in incremental sheet metal forming processes, where there are very large local unit pressures and friction-assisted heating [9,10].

In the stamping process, there are many areas where friction forces of different values occur. In general, friction is an undesirable phenomenon in the blankholder zone and at the edge of the draw die. The highest local contact pressures occur at the edge of the draw die. The sheet metal in this area is subject to large deformations. However, the friction of the sheet metal against the punch surface plays a positive role because it increases the force, causing cracking of the wall of drawpiece. High friction in the blankholder zone causes excessive thinning of the sheet metal, and sometimes the flow of material from the blankholder zone to the wall of drawpiece may be blocked.

Lubricating the friction pair is effective and is the cheapest way to reduce friction [11]. Due to their consistency, solid-based lubricants, emulsions and liquid lubricants (oils) are distinguished [12]. According to the criterion of origin, lubricants are divided into refined (obtained from crude oil), synthetic, and natural lubricants (vegetable oils and animal fats). Recently, appropriate surface preparation to ensure the creation of lubricating pockets that retain the lubricant during the friction process has become increasingly important [13]. The basic texturing techniques for metal forming tools include hammering [14], laser texturing [15], electrical discharge machining [16] and burnishing [17]. Friction reduces tool life by wearing it out. An effective way to improve the wear resistance of sheet metal forming tools is to also apply wear-resistant coatings using chemical or physical methods, heat or thermochemical treatment, and mechanical surface-strengthening methods, such as burnishing.

The most important property of the lubricant from the point of view of its use in plastic forming processes is its viscosity [18]. The lubricant should also effectively separate rubbing surfaces so as to minimise or eliminate the mechanical interaction of the tool with the sheet metal surface roughness. In processes occurring at elevated temperatures, the lubricant should be resistant to high temperatures and have stable physicochemical properties over a wide temperature range [19]. During sliding, the surface fractures via the tearing of the material under an intense thermal setting [20].

Due to their high availability and production traditions, petroleum-based lubricants are widely used. Petroleum-based lubricants typically used in sheet metal forming introduce large amounts of pollutants into the environment [21]. Currently, for ecological and legal reasons [22], the tendency is to form sheets either with a small amount of lubricant, without using a lubricant, with a minimum quantity of lubrication (MQL), or with an environmentally friendly lubricating coolant [23]. Another approach is to use vegetable-based biolubricants. These include edible and non-edible lubricants. Due to the long-chain fatty acids and polar end groups, the structure of vegetable oil is amphiphilic. Vegetable oils have better intrinsic boundary lubricant properties due to the presence of these long-chain fatty acids [24]. Biolubricants are characterised by high lubricity, a high flash point and low evaporation losses [25]. Vegetable oil-based lubricants are non-toxic and react less with chemicals, water and iron alloys than mineral oils [26].

Currently, many research groups have focused on the use of vegetable oils in metal forming operations. Bio-based lubricating oils have been proven to be competent compared to conventional petroleum-based oils [27]. Rapeseed, soybean, sunflower, peanut, coconut, linseed and many other oils are considered potential biolubricants [28]. Więckowski et al. [29] and Adamus et al. [30] proposed replacing conventional lubricants with ones based on boric acid and vegetable oils. Więckowski and Dyja [31] investigated the efficiency of some vegetable oils in reducing friction when forming titanium sheets prone to the galling phenomenon. They concluded that the use of popular vegetable oils (sunflower, rapeseed and olive oil) allowed for a reduction in friction for a steel–titanium friction pair. Adamus et al. [32] developed environmentally friendly methods for applying H3BO3 to a metal sheet by spraying it onto a thin layer of rapeseed oil that had been previously applied to the metal sheet surface. Trzepieciński et al. [33] tested the lubrication efficiency of non-edible oils with the most frequently tested edible oils (sunflower and rapeseed) in a steel-steel contact pair. It was found that the lubrication efficiency of the oils tested depends on the contact pressure and deformation of the sheet metal. Szewczyk and Szwajka [34] investigated the tribological performance of non-edible (Moringa and Karanja) and edible (sunflower and rapeseed) oils in a flat-die strip drawing test. It was found that non-edible oils achieved the lowest values of the coefficient of friction. Carcel et al. [24] investigated the performance of some of these vegetable oils (without additives) during the stamping of car body parts.

The authors confirmed a reduction in the coefficient of friction of steel sheets lubricated with vegetable oils (corn, sunflower and soybean) compared to those lubricated with mineral oil. Moreover, the effective anticorrosion protection provided by vegetable oils was confirmed (16 weeks without red rust when applied onto bare steel sheets). Shashidhara and Jayaram [35] used non-edible oils (Pongam and Jatropha) in the deep drawing of 304L stainless steel sheets. The tested lubricants ensured better material flow/draw-in-length and uniform thickness profiles compared to mineral oil lubrication. Nurul et al. [36] investigated palm oil-based lubricants in cold extrusion processes. The palm olein and palm kernel oil showed an enhanced extrusion load and no severe wear on the product surface. Reviews of recent research and the properties of vegetable oil-based biolubricants have been published by Aiman et al. [37] and Woma et al. [38].

There is little research on the use of non-modified vegetable-based lubricants in the sheet metal forming of deep drawing high-quality steel sheets. For this reason, this article focuses on testing the lubrication efficiency of typical vegetable oils using a special tribotester intended for assessing friction in the deep drawing process. The coefficient of friction is a quantitative indicator of the resistance resulting from the frictional interaction of the sheet metal surface and the tool surface. Knowledge of the value of the coefficient of friction is necessary when designing technology for the forming of body parts. Friction is also responsible for the quality of the product’s surface. Steel sheet body panels are covered with anticorrosion coatings, which requires a specific quality of the substrate. So, it is necessary to ensure similar quality of the entire surface of body panels. The largest relative displacements of the sheet metal on the tool surface occur in the blankholder zone. So, knowing the friction conditions in the blankholder zone is particularly important. Due to the influence of lubricant properties and friction process parameters on the coefficient of friction value, which is difficult to analytically assess, multi-layer neural networks (MLNNs) were used to process the test data. Many architectures and training algorithms were used to build the optimal MLNN model.

## 2. Materials and Methods

### 2.1. Test Material

The test material was hard-rolled low-carbon 1.0338 (DC04) steel sheets (Centrostal S.A., Warszawa, Poland) with a thickness of 0.8 mm. Steel 1.0338 consists mainly of iron, with the addition of C (max. 0.08 wt.%), S (max. 0.02 wt.%), P (max. 0.025 wt.%), Mn (0.40 wt.%), Si (0.10 wt.%) and Al (0.015–0.07 wt.%). The mechanical properties of the sheets were determined using a uniaxial tensile test. A Zwick/Roell Z100 testing machine (Ulm, Germany) was used to determine the basic mechanical properties of the sheet metals according to the EN-ISO 6892-1 standard. In addition to the typical mechanical properties, the strain hardening parameters were determined based on the approximation of the true stress–strain relationship according to the power law function:

σ = K × ε^n^ (1)

where σ is the yield strength, K is the strength coefficient, ε is the true plastic strain and n is the strain hardening coefficient.

**Figure 1:**
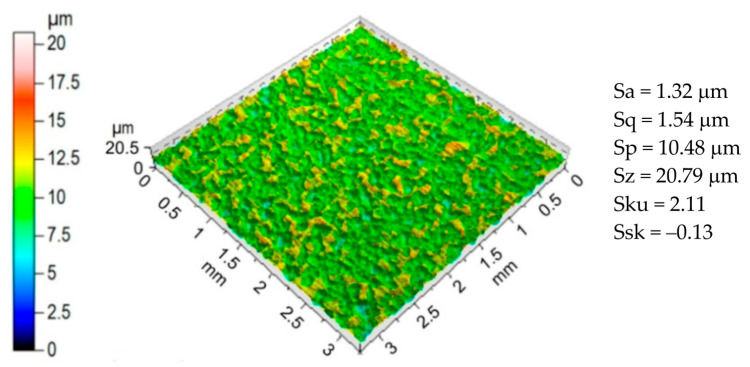
The surface topography and basic surface roughness parameters of a 1.0338 steel sheet.

Three specimens were tested and the following parameters were determined: yield strength, 185 MPa; ultimate tensile stress, 302 MPa; total elongation, 22%; K = 481 MPa; n = 0.18. The surface topography of the sheets (Figure 1) was analysed using a CCI Lite 3D scanning profilometer. The basic parameters characterizing the sheet surface were determined as follows: Sa, Sq, Sp, Sz, Sku and Ssk (Figure 1).

### 2.2. Friction Test Procedure

The friction conditions presented in this article concern the blankholder zone in the deep drawing process. The strip drawing test, which involves pulling a strip of sheet metal between two fixed countersamples, was used to determine the value of the coefficient of friction. The countersamples were made from 1.2063 (145Cr6) tool steel. The strip drawing test is a basic test for assessing friction conditions in sheet metal forming [39,40].

**Figure 2:**
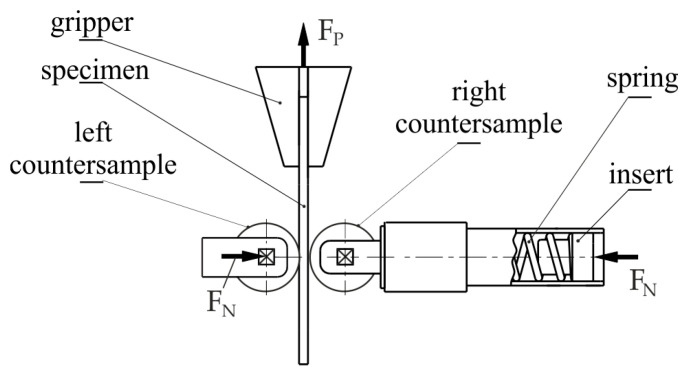
Schematic diagram of the friction tester (F_P_—pulling force, F_N_—normal force).

**Figure 3:**
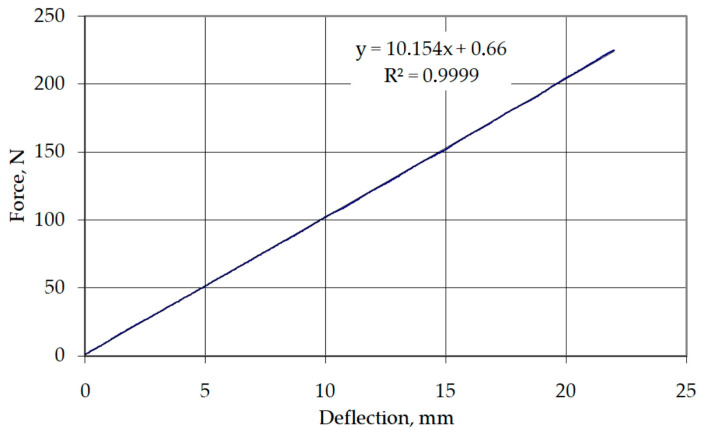
The force-deflection curve of the spring.

The developed friction tester consisted of two rounded countersamples (Figure 2) between which a strip of sheet metal with a width of w = 0.018 m moved during the test. The upper end of the sample was mounted in the holder of the Zwick/Roell Z100 testing machine. The normal force was set through a spring with known force-deflection characteristics (Figure 3). During the test, the normal force was increased to obtain contact pressure values ranging between approximately 1.7 and 5 MPa. This was the range used in the blankholder zone in the deep drawing process [41,42].

The value of the pulling force of the sheet metal strip was measured using the testing machine’s measuring system. Based on the normal force F_N_ and the pulling force F_P_, the average value of the coefficient of friction for a friction distance of approximately 40 mm was determined according to the following relationship:

(2) $$\mu =\frac{F_{P}}{{2F}_{N}}$$

Based on the normal force value, the contact pressure was determined based on the following relationship proposed by Ter Haar [43]:

(3) $$p=\frac{\pi }{4}\sqrt{\frac{F_{N}/wE}{2\pi R}}$$

where R is the radius of the countersample and E is the combined Young’s modulus of the sheet material:

(4) $$E=\frac{2E_{1}E_{2}}{E_{1}\left(1-\nu_{2}^{2}\right)+E_{2}\left(1-\nu_{1}^{2}\right)}$$

where E_1_ and E_2_ are the Young’s moduli of the sheet metal and countersample materials, respectively, and ν_1_ and ν_2_ are the Poisson’s ratios of the sheet metal and countersample materials, respectively.

The following values were used in the calculations: E_1_ = 186.9 GPa, E_2_ = 210 GPa [44], ν_1_ = ν_2_ = 0.3.

The research results presented in this work are limited to the sliding speed constant. The experiments were carried out at a constant speed of 5 mm·s^−1^ and at room temperature. The tests were carried out under dry friction conditions and using edible (corn, sunflower, rapeseed) and inedible (karanja, moringa) vegetable oils. Both sides of the specimen were oiled using a roller system. A uniform oil coating between 1.5 and 2 g·m^−2^ [24] was applied. The basic physical properties of the oils are presented in Table 1.

Three sets of countersamples with different surface roughness values were tested. The measurement of basic surface roughness parameters of countersamples was carried out using the Alicona InfiniteFocus instrument. The average surface roughness of the countersamples was Sa = 0.44 μm, Sa = 0.56 μm and Sa = 1.34 μm. The values of the Sa parameter correspond to the average surface roughness measured along the forming cylindrical samples: Ra = 0.32 μm, Ra = 0.63 μm and Ra = 1.25 μm. The basic surface roughness parameters of the countersamples are listed in Table 2.

### 2.3. Artificial Neural Networks

**Figure 4:**
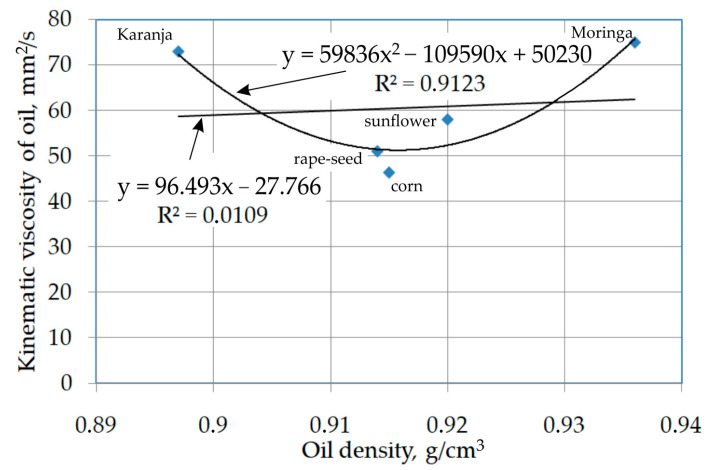
The relationship between the density and viscosity of the tested oils.

MLNNs, also known as multi-layer perceptrons (MLPs), were used to analyse the complex relationships between the parameters of the friction process under lubrication conditions with edible and non-edible oils and the value of the coefficient of friction. The Statistica program was used to create and analyse the MLNNs. Neural networks with one hidden layer were used because according to Hertz et al. [46], three-layer neural networks are capable of analysing any non-linear task. The average roughness of the countersamples, the contact pressure and the physical parameters of the oil were selected as input parameters. Oils with a similar viscosity may have significantly different densities. Kinematic viscosity is the most frequently chosen parameter to assess the effectiveness of sheet metal lubrication. The potential synergistic effects of density and oil viscosity on friction under sheet metal forming conditions have not been explored so far. Taking into account (i) the non-linear relationship between the kinematic viscosity and the density of the lubricants used (Figure 4) and (ii) the potential synergistic simultaneous effects of kinematic viscosity and oil density, which are difficult to analytically assess, we decided to initially introduce both of these parameters into the network input.

Normalisation of the test data is the process by which all input data are reduced to the intervals [0, 1] or [−1, 1]. If normalisation is not performed, there is a possibility of premature overfitting of the network. In the case of neural networks, the data normalisation operation is mandatory. The data should be normalised in such a way that the new values have the same distribution with respect to the minimum and maximum values of the raw experimental data.

The min–max function (Equation (5)) was used to normalise the data, which allowed the values of the input parameters and the output parameter to be transformed into the range [0, 1]. In fact, normalisation modifies the value of the data, but it achieves this while maintaining information about the distance between each point:

(5) $$N=\frac{X-x_{min}}{x_{max}-x_{min}}$$

where X is the value of the normalised parameter and x_min_ and x_max_ are minimum and maximum values of the normalised parameter, respectively.

The entire training set contained 120 sets of input parameters and the corresponding value of the coefficient of friction. From this set, a validation set (15% of the training set) and a test set (15% of the training set) were randomly separated. The remaining data constituted the training set. The weights of each neuron were configured during the training phase. The effectiveness of neural network training was checked using various algorithms: classical back propagation, conjugate-gradients, quasi-Newton, quick propagation and Levenberg–Marquardt. The value of the deviation between the expected output value and the predicted value was taken as an indicator of network quality. At the beginning of the training process, the error value was very high because the neuron weights were randomly generated and gradually decreased during the training process. The weights of neurons that contributed to generating the significant error were modified more significantly than the weights that contributed to the marginal error.

### 2.4. Sensitivity Analysis

The possible significance of the effect of density on the coefficient of friction was assessed using sensitivity analysis. If the lack of influence of oil density on the coefficient of friction value was confirmed, this parameter was removed from the analysis. The MLNN output parameter was the coefficient of friction.

## 3. Results and Discussion

### 3.1. Effectiveness of Lubrication

The effectiveness of lubrication (EoL) was estimated on the basis of the percentage difference between the coefficient of friction determined under dry friction conditions and under lubrication with a specific type of oil:

(6) $$EoL=\frac{\mu_{mean}(dry\;friction)-\mu_{mean}(lubricated\;conditions)}{\mu_{mean}(dry\;friction)}\times 100\%$$

**Figure 5:**
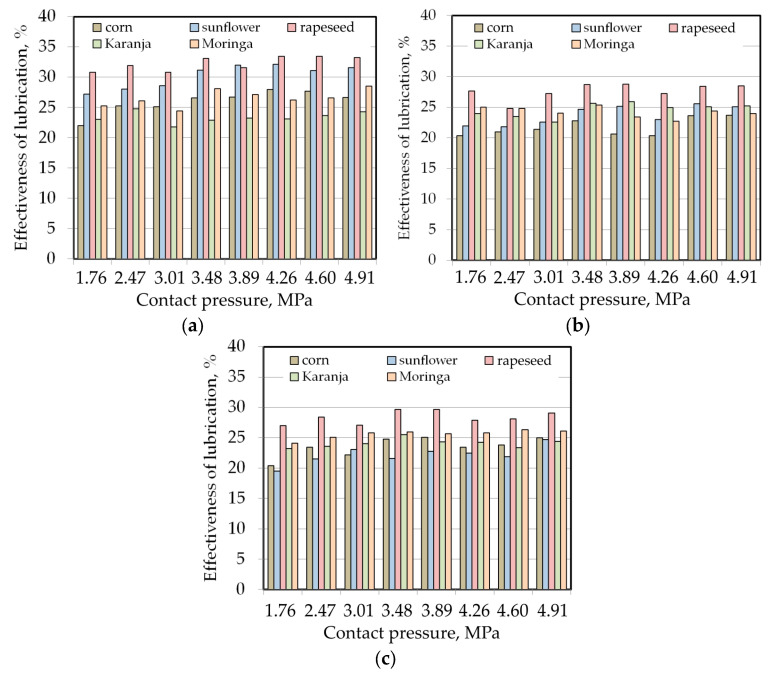
Lubrication efficiency determined during friction testing with countersamples with different average roughness values: (**a**) Sa = 0.44 μm, (**b**) Sa = 0.56 μm and (**c**) Sa = 1.34 μm.

During friction testing with steel countersamples with an average surface roughness of Sa = 0.44 μm (Figure 5a), sunflower and rapeseed oils were characterised by the highest lubrication efficiency (approximately 27–32%). However, rapeseed oil had better lubricating properties than sunflower oil. A slight tendency to increase the lubrication efficiency of these oils with increasing contact pressure was observed. Karanja oil had the least impact on reducing the coefficient of friction. Moreover, in the entire range of applied contact pressures, the EoL index for this oil was the most stable in the range of 21.8–24.7%. The difference between the lubrication efficiency of the tested oils at an analysed pressure range varied from 7.16% for a contact pressure of 2.47 MPa to 10.3% for a contact pressure of 4.26 MPa.

Increasing the average surface roughness of the countersamples from Sa = 0.44 μm to Sa = 0.56 μm resulted in a decrease in the lubrication efficiency of the tested oils, except for moringa oil, for which a slight 0.7–2.7% improvement in lubrication efficiency was observed (Figure 5b). Increasing the surface roughness of the countersamples provides larger spaces in the valleys between the surface asperities where the lubricant can be retained. At the same time, with high roughness of the rubbing surfaces, it is more difficult to create closed lubricant pockets. Under the influence of contact pressure, the summits of the asperities deform, increasing the pressure of the lubricant trapped in the valleys. Oil under pressure transfers part of the load, reducing the pressure acting on the surface asperities [47].

The highest lubrication efficiency presented by rapeseed oil does not exceed 28.7%. In contrast, in the entire range of analysed contact pressures, corn oil showed the worst lubricating properties (EoL between 20.3% for a contact pressure of 4.26 MPa and 23.7% for a contact pressure of 4.91 MPa). In the contact pressure range between 1.76 and 3.01 MPa, moringa oil was the second most effective lubricant. At pressures greater than 3.01 MPa, Karanja oil took second place. Despite the increase in the average roughness of the countersamples from Sa = 0.56 μm to Sa = 1.34 μm, no significant change in the EoL index was observed (Figure 5c). Within the entire range of contact pressures tested, rapeseed oil again showed beneficial friction-lowering properties. As can be seen in Figure 5, it is difficult to draw qualitative or quantitative conclusions representing all the lubricants tested. Therefore, artificial neural networks will be used to recognise the relationship between the friction process parameters and friction. The results of this research are presented in the next section.

### 3.2. Artificial Neural Networks

Many analyses have been performed with different network architectures and training algorithms. The root mean square (RMS) error is considered an excellent general purpose error metric for neural networks predictions [48,49]. The RMS error is the square root of the mean of the square of the total error [50]:

(7) $$RMS\;error=\sqrt{\frac{1}{N}\sum_{i=1}^{N}\mu_{i}-\vartheta_{i}^{2}}$$

where μ_i_ is the i-th predicted value of the coefficient of friction and $\vartheta_{i}$ is the i-th observation and n is the number of observations.

**Figure 6:**
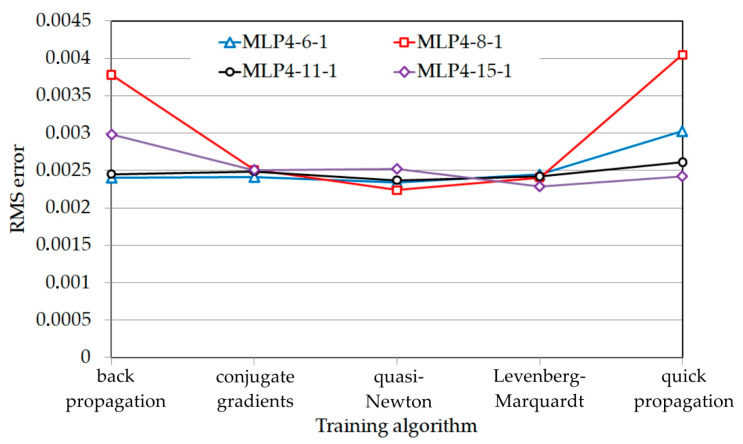
The effect of the training algorithm on the value of the RMS error for the test set.

Figure 6 shows the impact of the training algorithm and the number of neurons in the hidden layer on the RMS error value for the data from the test set that did not participate in the training process. It was observed that the conjugate gradient algorithm, quasi-Newton algorithm and Levenberg–Marquardt algorithm showed similar levels of effectiveness for the process of training networks with different architectures. However, the lowest RMS error value was observed for the MLP-4-8-1 network trained with the quasi-Newton algorithm. Moreover, the quick propagation learning algorithm is not effective for networks with a small number of neurons in the hidden layer. For this algorithm, the more neurons in the hidden layer, the greater the predictive accuracy of the network.

Sensitivity analysis allows important variables to be distinguished from those that do not affect the predictive quality of the artificial neural network. Input variables are usually not independent. Sensitivity analysis shows the loss incurred when a specific variable is rejected. If two or more variables contain the same information, the rejection of one of the variables does not reduce the quality of the network because the information is available in another variable or variables. However, if all the correlated variables are removed, the network is deprived of a very important information carrier.

Table 3, Table 4, Table 5, Table 6 and Table 7 present the results of the sensitivity analysis of the considered neural network architectures. The basic measure of network sensitivity is the quotient of the error (‘Ratio’ in Table 3, Table 4, Table 5, Table 6 and Table 7) obtained when starting the network for a dataset without one variable and the error obtained with the full set of variables. The greater the error after rejecting a variable in relation to the original error, the more sensitive the network is to the absence of this variable [51]. If the ‘Ratio’ ≤ 1, removing the variable has no impact on the network quality. The ‘Error’ in Table 3, Table 4, Table 5, Table 6 and Table 7 means the total error of the network after removing a specific variable from the dataset. Variables can be ranked in order of importance. The higher the ‘Rank’ value in Table 3, Table 4, Table 5, Table 6 and Table 7, the greater the importance of a specific variable. 

Regardless of the MLNN architecture, the sensitivity analysis of networks trained using the classical back propagation algorithm, the conjugate gradients algorithm, the quasi-Newton algorithm and the Levenberg–Marquardt algorithm showed that the kinematic viscosity of the oil was the most important parameter. In general, the least important parameter was contact pressure. For networks trained using the classical back propagation algorithm (Table 3) and the conjugate gradients algorithm (Table 4), the second most important parameter is the average surface roughness of the countersamples, and oil density is the third most important parameter. The average surface roughness of the countersamples and the oil density are the second and third most important parameters in MLNN models trained with the quasi-Newton algorithm (Table 5) and Levenberg–Marquardt (Table 6) algorithm. The most difficult to analyse are the results of the sensitivity analysis of the networks trained using the quick propagation algorithm (Table 7), because the ranking of variables strongly depends on the architecture of the neural network, and more precisely on the number of neurons in the hidden layer. Nevertheless, the kinematic viscosity of the oil and the average surface roughness of the countersamples are generally the variables that provide the most important knowledge at the input of the neural network. Contact pressure is considered as the third parameter in order of importance for all the network architectures.

The MLP-4-8-1 network model trained using the quasi-Newton algorithm (Figure 6), characterised by the smallest network error for the test set, was selected for further analysis. Selected regression statistics for this network are presented in Table 8. The highest correlation value concerns the training set because this contained the large majority of the test data. The higher the test correlation, the higher the prediction accuracy. The ratio of the standard deviation of errors and the standard deviation of the value of the explained variable (S.D. ratio) is an additional indicator of network quality. This value should be less than 1. The lower this value, the higher the predictive quality of the network.

**Figure 7:**
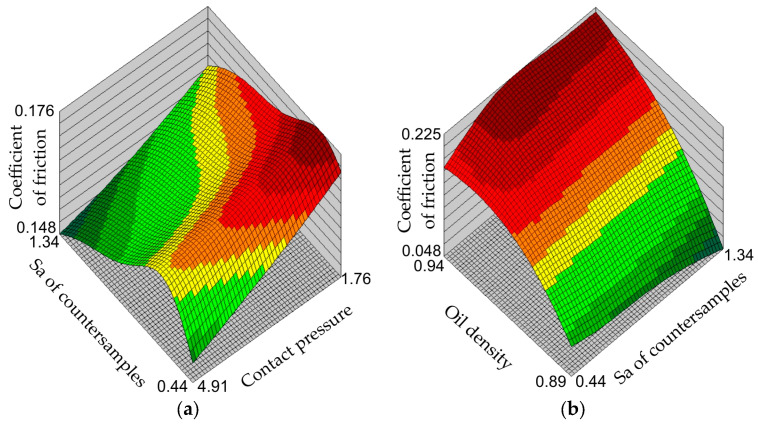
Response surfaces of the MLP-4-8-1 network showing the effect of (**a**) the surface roughness of the countersamples and contact pressure and (**b**) the surface roughness of countersamples and oil density on the value of coefficient of friction.

**Figure 8:**
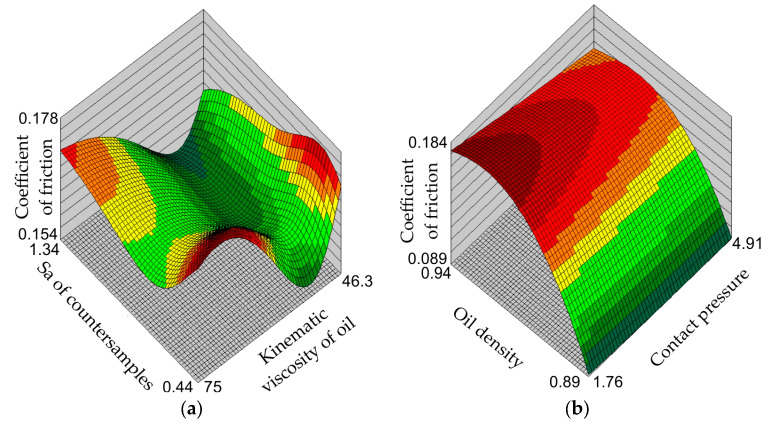
Response surfaces of the MLP-4-8-1 network showing the effect of (**a**) the surface roughness of the countersamples and the kinematic viscosity of the oil and (**b**) the contact pressure and oil density on the value of coefficient of friction.

**Figure 9:**
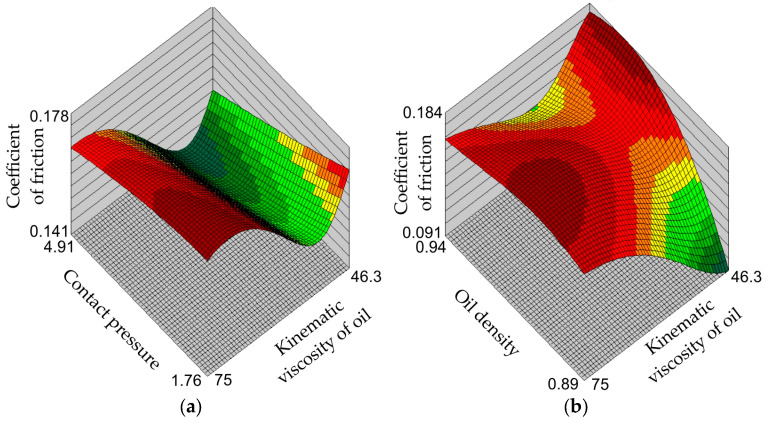
Response surfaces of the MLP-4-8-1 network showing the effect of (**a**) the contact pressure and the kinematic viscosity of the oil and (**b**) the kinematic viscosity of oil and oil density on the value of coefficient of friction.

Figure 7, Figure 8 and Figure 9 show the response surfaces of the MLP-4-8-1 network. The response surfaces of the MLNN represent the values of the coefficient of friction for selected combinations of input parameters. Due to the limitations of three-dimensional graphs, response surfaces were prepared for two input parameters and one output parameter (coefficient of friction). The remaining values of input parameters, not presented in a specific graph, assume average values.

Increasing the contact pressure reduces the coefficient of friction. This is a phenomenon observed in sheet metal forming consisting of a non-linear relationship between the contact force and the friction force, as also observed by Dou and Xia [52] and Vollertsen and Hu [53]. The nature of interaction between the asperities of the ‘hard’ tool and the summits of the asperities of the relatively ‘soft’ sheet metal significantly changes the contact conditions (Figure 7a). According to the Edwards and Halling [54], when the interacting surfaces have low roughness values, the coefficient of friction is relatively small and then increases with the increase in surface roughness by intensifying the mechanical cooperation mechanism of the surface asperities. With a further increase in surface roughness, the share of mechanical interpenetration of the asperity summits decreases, the share of the adhesion mechanism decreases and the effect of separating the rubbing surfaces increases as a result of the lubricant cushion effect. Under these conditions, friction decreases with increasing roughness. With an increase in contact pressure, the share of mechanical interpenetration of the asperity summits decreases, the share of the adhesion mechanism decreases and the effect of separating the rubbing surfaces increases as a result of the lubricant cushion effect. Increasing surface roughness increases the volume between the surface asperities that can accommodate the lubricant. As a result of contact pressure, a specific lubricating cushion is created [55,56]. Due to the increasing resistance to movement of the lubricant layers with increasing oil density, the value of the coefficient of friction increases (Figure 7b and Figure 8b).

The influence of kinematic viscosity on the value of the coefficient of friction is strongly dependent on the average surface roughness of the countersamples (Figure 8a). The viscosity of a liquid is the result of internal friction forces between the liquid molecules. Highly viscous oil fills the surface roughness valleys with greater resistance. However, the higher the viscosity of the oil introduced into the friction pair, the more difficult it is to squeeze it out from contact zone and the greater the internal resistance to friction. At a certain value of the kinematic viscosity of the oil, there is an area with a reduced coefficient of friction value (Figure 8a and Figure 9a). This is the area separating the two main friction mechanisms. The first, occurring with low roughness of the contacting surfaces, is due to adhesion and the limited influence of the lubricant on friction. In contrast, with high surface roughness, the phenomenon of adhesive contact is very limited. However, in correlation with high pressure, there is a strong interaction of the high summits of the tool surface asperities with the sheet metal surface (roughening). The summits of the sheet metal asperities, unlike the tool surface, are subject to strong plastic deformation and the occurrence of flattening.

The character of the influence of the kinematic viscosity of the oil on the value of the coefficient of friction is the same regardless of the contact pressure value (Figure 9a)—that is, at each contact pressure value, the coefficient of friction value first decreases to the minimum value and then increases until it reaches the maximum value. From the point of view of the conditions of the experimental tests, the most advantageous reduction in the coefficient of friction value can be achieved by using a grease which has both low viscosity and low density (Figure 9b). These conditions are met by rapeseed and corn oils (Figure 4). It is also unfavourable to use oils with high kinematic viscosity under the pressure conditions considered in the tests.

## 4. Conclusions

In this article, the lubrication efficiency of typical vegetable oils was experimentally tested using a strip drawing test intended for assessing friction in sheet metal forming processes. The research used a special strip drawing tribometer, which allowed us to determine the value of the coefficient of friction in the blankholder zone in the deep drawing process. Due to the trend towards sustainable production, vegetable oils were used in this research work as an alternative to petroleum-based lubricants. The research results may constitute the basis for further research on other oils of natural origin. Due to the complex relationships between the conditions of the friction process and the value of the coefficient of friction, artificial neural networks belonging to artificial intelligence methods were used. MLNNs with different architectures and training methods were used to assess the effect of the friction process parameters on the coefficient of friction value. The main research conclusions are as follows:

- Rapeseed oil provided the best lubrication efficiency during friction testing for all tested countersamples with an average surface roughness in the range of 0.44–1.34 μm. Depending on the parameter Sa of the countersamples, rapeseed oil reduced the coefficient of friction by 30.8–33.4% (Sa = 0.44 μm), 24.7–28.7% (Sa = 0.56 μm) and 27.0–29.6% (Sa = 1.34 μm), compared to dry friction conditions. Rapeseed oil had a viscosity and density similar to that of corn oil. The viscosities of these oils were the lowest among all the oils tested. However, the effectiveness of lubrication of these two oils was different. This difference may partly be explained by the different chemical compositions and as a result of diverse physico-chemical properties which were not investigated in this paper. According to Santos et al. [57], rapeseed oil contains 65% monosaturated fatty acids, 29% polysaturated fatty acids and 5% saturated fatty acids. Meanwhile, corn oil contains completely different proportions of fatty acids: 34% monosaturated fatty acids, 50% polysaturated fatty acids and 16% saturated fatty acids.
- Karanja oil had the least impact on reducing the coefficient of friction during friction testing with countersamples with an average roughness of Sa = 0.44 μm. During friction testing with countersamples with the highest considered roughness, sunflower oil was the least effective in reducing the coefficient of friction value.
- Among all the analysed MLNN architectures, the lowest RMS error value was observed for the MLP-4-8-1 network trained with the quasi-Newton algorithm. This network is also characterised by a high correlation value for the test set (R^2^ = 0.9619) and the validation set (R^2^ = 0.9599). The effectiveness of MLNN training using the classical back propagation and quick propagation algorithms was the most sensitive to the architecture of the MLNN. For all network architectures trained with the Levenberg–Marquardt, quasi-Newton and conjugate gradients algorithms, the value of the RMS error for the training set was similar.
- For most of the analysed networks trained using the various algorithms, the kinematic viscosity of the oil was shown to be the most important parameter in assessing the friction phenomenon of the sheets tested. In general, for networks trained using the classical back propagation, conjugate gradients, quasi-Newton and Levenberg–Marquardt algorithms, contact pressure was the parameter that least affects the value of the CoF in the range of contact pressures considered (about 1.7–5 MPa).
- Based on the response surfaces of the MLP-4-8-1 network, it was found that as contact pressure increases, the coefficient of friction tends to decrease slightly. The influence of kinematic viscosity on the value of the coefficient of friction is strongly dependent on the average surface roughness of the countersamples. At low roughness values of the interacting surfaces, the coefficient of friction increases with the increase in surface roughness by intensifying the mechanical cooperation mechanism of the surface asperities. With a further increase in surface roughness, the share of mechanical interpenetration of the asperity summits and adhesion mechanism decreases, and under these conditions, friction decreases with increasing roughness.

## Figures and Tables

**Table 1:** Kinematic viscosity and density of oils tested, prepared on the basis of [33,45].

Oil	Kinematic Viscosity η_k_, mm^2^/s	Density, g/cm^3^
corn	46.3	0.915
sunflower	58	0.92
rapeseed	51	0.914
Karanja	73	0.897
Moringa	75	0.936

**Table 2:** Basic surface roughness parameters of countersamples.

Set of Countersamples	Sa, μm	Sq, μm	Sz, μm	Sp, μm	Sku	Ssk
Set_1	0.44	0.56	69.9	17.9	8.43	−0.93
Set_2	0.56	0.72	22.04	15.6	4.11	−0.27
Set_3	1.34	1.57	51.6	11.1	3.01	−0.29

**Table 3:** The results of the sensitivity analysis of the input parameters for MLNNs trained using the classical back propagation algorithm.

MLNN	Parameter	Average Surface Roughness Sa of Countersamples	Contact Pressure	Oil Density	Kinematic Viscosity of Oil
MLP-4-6-1	Rank	2	4	3	1
	Error	0.01227	0.004308	0.006065	0.02117
	Ratio	6.935904	2.436233	3.429362	11.97275
MLP-4-8-1	Rank	2	4	3	1
	Error	0.01403	0.004716	0.007152	0.01621
	Ratio	4.76152	1.600634	2.427241	5.503119
MLP-4-11-1	Rank	2	3	4	1
	Error	0.0108	0.004567	0.003528	0.01233
	Ratio	5.641023	2.385629	1.842975	6.438527
MLP-4-15-1	Rank	2	4	3	1
	Error	0.01177	0.004361	0.00769	0.02263
	Ratio	6.087303	2.255849	3.977502	11.70501

**Table 4:** The results of the sensitivity analysis of the input parameters for MLNNs trained using the conjugate gradients algorithm.

MLNN	Parameter	Average Surface Roughness Sa of Countersamples	Contact Pressure	Oil Density	Kinematic Viscosity of Oil
MLP-4-6-1	Rank	2	4	3	1
	Error	0.01458	0.004276	0.01378	0.03424
	Ratio	9.265555	2.717773	8.760405	21.765
MLP-4-8-1	Rank	2	4	3	1
	Error	0.012	0.004303	0.008007	0.02287
	Ratio	8.469641	3.0368	5.651413	16.14482
MLP-4-11-1	Rank	2	4	3	1
	Error	0.01518	0.004279	0.01391	0.03558
	Ratio	10.61238	2.991009	9.722983	24.86929
MLP-4-15-1	Rank	2	4	3	1
	Error	0.0118	0.004437	0.008098	0.02074
	Ratio	5.383217	2.025121	3.69574	9.466121

**Table 5:** The results of the sensitivity analysis of the input parameters for MLNNs trained using the quasi-Newton algorithm.

MLNN	Parameter	Average Surface Roughness Sa of Countersamples	Contact Pressure	Oil Density	Kinematic Viscosity of Oil
MLP-4-6-1	Rank	3	4	2	1
	Error	0.01293	0.004238	0.03852	0.05884
	Ratio	7.718678	2.530221	22.99976	35.13553
MLP-4-8-1	Rank	2	4	3	1
	Error	0.01012	0.004237	0.008161	0.02054
	Ratio	6.766078	2.831611	5.454082	13.72464
MLP-4-11-1	Rank	2	4	3	1
	Error	0.01138	0.004261	0.006825	0.023346
	Ratio	7.545811	2.826716	4.527627	15.48629
MLP-4-15-1	Rank	3	4	2	1
	Error	0.01117	0.00428	0.017606	0.02113
	Ratio	8.300681	3.18103	13.08428	15.69999

**Table 6:** The results of the sensitivity analysis of the input parameters for MLNNs trained using the Levenberg–Marquardt algorithm.

MLNN	Parameter	Average Surface Roughness Sa of Countersamples	Contact Pressure	Oil Density	Kinematic Viscosity of Oil
MLP-4-6-1	Rank	3	4	2	1
	Error	0.01382	0.004281	0.02016	0.036513
	Ratio	9.054658	2.805358	13.21027	23.92837
MLP-4-8-1	Rank	2	4	3	1
	Error	0.01097	0.004295	0.006834	0.01921
	Ratio	7.587761	2.96978	4.725448	13.27895
MLP-4-11-1	Rank	3	4	2	1
	Error	0.01127	0.004293	0.01614	0.03304
	Ratio	8.256395	3.146023	11.82993	24.2113
MLP-4-15-1	Rank	3	4	2	1
	Error	0.01175	0.004326	0.01962	0.029
	Ratio	9.020841	3.321519	15.06749	22.26404

**Table 7:** The results of the sensitivity analysis of the input parameters for MLNNs trained using the quick propagation algorithm.

MLNN	Parameter	Average Surface Roughness Sa of Countersamples	Contact Pressure	Oil Density	Kinematic Viscosity of Oil
MLP-4-6-1	Rank	2	3	4	1
	Error	0.005431	0.005089	0.003553	0.007181
	Ratio	1.594428	1.494238	1.043223	2.108318
MLP-4-8-1	Rank	4	3	1	2
	Error	0.005774	0.005978	0.008271	0.006978
	Ratio	1.272161	1.317044	1.82234	1.537503
MLP-4-11-1	Rank	2	3	4	1
	Error	0.006119	0.004272	0.002575	0.007295
	Ratio	4.045816	2.824535	1.702513	4.823144
MLP-4-15-1	Rank	1	3	4	2
	Error	0.006312	0.004435	0.003386	0.006275
	Ratio	3.960661	2.783153	2.124575	3.93749

**Table 8:** Regression statistics for the MLP-4-8-1 network trained using the quasi-Newton algorithm.

Parameter	Training Set	Validation Set	Test Set
Absolute error mean	0.001252	0.001832	0.001807
S.D. ratio	0.1866378	0.2819774	0.3003911
Correlation	0.9824294	0.9599284	0.961962

## Data Availability

Data are contained within the article.
